# A novel use of colonoscopy forceps for biopsy of intervertebral disc

**DOI:** 10.1308/003588413X13511609958055e

**Published:** 2013-03

**Authors:** MD Wijeratna, AJ Cook, J Powell

**Affiliations:** Ipswich Hospital NHS Trust, UK

## Background

Core biopsy of vertebral bone is traditionally carried out in the prone position under radiological guidance using a trephine. If biopsy of the intervertebral disc is required, the same technique and instrumentation is used or fine-needle aspiration is performed. The biopsy obtained with the trephine relies on capillary action and the volume of sample tissue is often small. The sensitivity of this technique is poor, with rates ranging between 42% and 62%.^1^ With a larger sample of tissue, it is probable that the sensitivity of the technique may be improved. We propose a novel technique (using equipment already present in the operating theatre) that increases the volume of sample tissue obtained and could improve the likelihood of an organism being isolated.

## Technique

**Figure 1:**
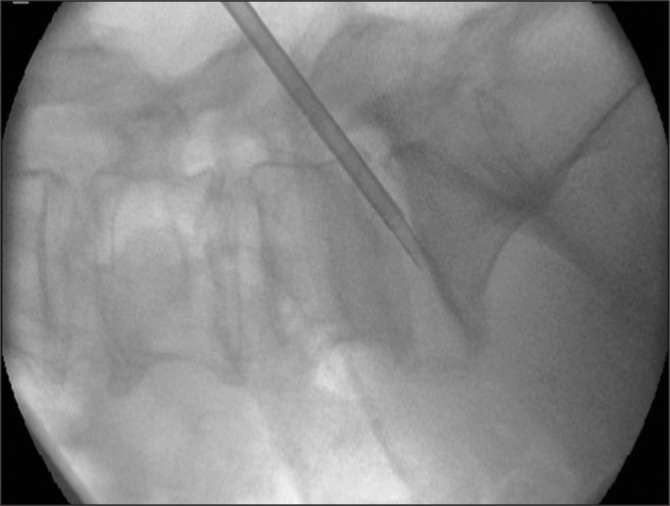
Intra-operative fluoroscopy of traditional core biopsy trephine

**Figure 2:**
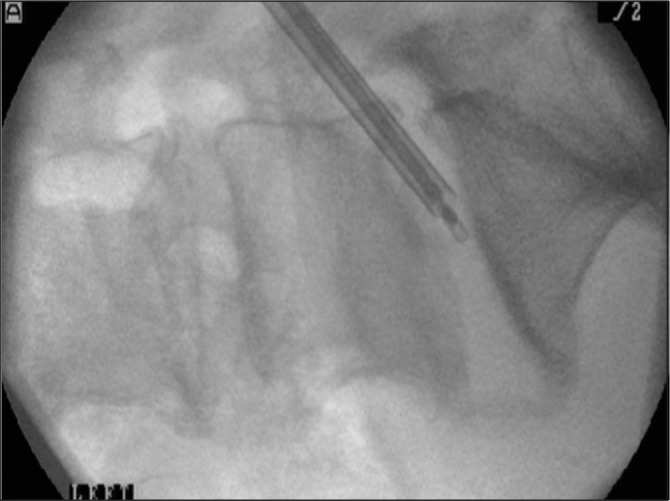
Intraoperative fluoroscopy of sampler with closed jaws

**Figure 3:**
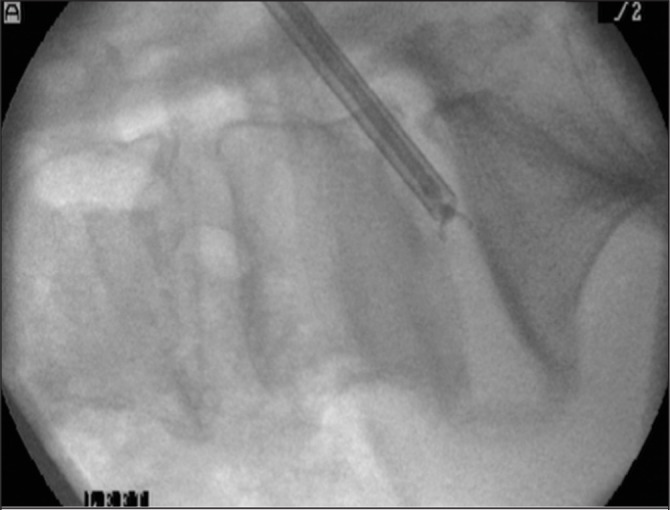
Intraoperative fluoroscopy of sampler with open jaws

The patient is placed prone and the core biopsy trocar is inserted under radiological guidance using standard techniques. A KyphX^®^ size 3 bone biopsy device trocar (Medtronic, Minneapolis, MN, US) has an inner diameter of 2.9mm. Instead of the trephine supplied in the kit (Fig 1), a colonoscopy biopsy forceps is passed down the trocar, and multiple samples are taken by opening and closing the jaws to remove tissue (Figs 2 and 3). The Radial Jaw^®^ 3 biopsy forceps (Boston Scientific, Natick, MA, US) has a 2.2mm opening jaw diameter and can be passed down a 2.8mm diameter working channel.

## Discussion

The use of a colonoscopy sampling device may increase the sensitivity of traditional core biopsy techniques to obtain a positive bacteriological culture or tissue type.
